# Time-bounded uncertainty in childhood cancer survivorship: a psycho-oncology perspective on result communication

**DOI:** 10.3389/fpsyg.2026.1890398

**Published:** 2026-08-26

**Authors:** Christian Prinz

**Affiliations:** Private Scholar, University of Oldenburg, Oldenburg, Germany

**Keywords:** childhood cancer survivorship, ecological momentary assessment, fear of cancer recurrence, health services research, patient portals, psycho-oncology, scanxiety, uncertainty

## Abstract

Follow-up after childhood cancer may be clinically routine, but it can still leave families in a poorly defined waiting period. A scan or blood test may be part of routine surveillance; however, the days that follow can feel far from ordinary. Families may not know when the results will arrive, who will interpret them, or whether silence has meaning. This Perspective proposes event-related distress windows (ERDWs) as an early service-design heuristic rather than a diagnosis or validated construct. The contribution is deliberately narrow and therefore testable: ERDW shifts attention from fear as content to waiting as a temporal form of care. It asks whether a modifiable result pathway concentrates uncertainty-related burden within a defined period. Bounded result communication can then be examined as a message condition that gives the interval a clear structure without adding harm. ERDW is useful only if it helps identify candidate pathways, specifies a minimal uncertainty mechanism, and frames a falsifiable service-level question. It should be discussed with survivors, parents, adolescents, multilingual families, and clinical teams before feasibility testing. If temporal concentration, mechanism, or safety cannot be shown, the frame should be narrowed or abandoned.

## Introduction

A scan or blood test may be medically ordinary but still disrupt family life while results are pending. For the service, it may be a small event; at home, it may become a waiting room without walls. Results may arrive tomorrow or next week, through a portal, phone call, or letter. Families may not know who will interpret them, what silence means, or when to ask for help. The clinical situation is familiar. The scientific question is narrow: can this interval be observed, measured, and ethically improved as a service-level signal?

Survivorship surveillance is protective. It can detect late effects, sustain trust, and reassure families that long-term care is still managed by a dedicated team (Bhakta et al., 2017; Landier et al., 2018; Marchak et al., 2022). The aim is not to reduce follow-up care but to ensure that any necessary waiting periods are more clearly defined. This Perspective uses event-related distress windows (ERDW) as a pre-co-design operational heuristic within uncertainty-in-illness, temporal-disruption, and medical-waiting traditions (Mishel, 1988; Charmaz, 1983; Timmermans and Buchbinder, 2010; Wongsomboon and Shepperd, 2022). The word “window” is deliberate. It refers to a time-limited observation period for service evaluation. It does not indicate a critical period, a vulnerability interval, a diagnosis, or a patient-facing label. The cited literature is therefore used to position adjacent concepts and the medical waiting problem. It is not used to claim that ERDW has already been validated.

The objective of this Perspective is therefore to define ERDW as a narrow service-design heuristic and to show how it can guide a first evaluation of result-communication pathways. This places the manuscript within psycho-oncology: it links a psychosocial burden—uncertainty while awaiting results—to a potentially modifiable feature of survivorship care, namely how services organize time, route, and explanation. The argument moves from the clinical problem to adjacent psycho-oncology constructs, candidate pathway criteria, bounded communication, evaluation, harms and equity, population differences, co-design, and conditional conclusions.

## ERDW as a heuristic, not a construct

The thesis is modest, and the evidentiary claims remain deliberately limited. Some follow-up result pathways may create a short period of uncertainty due to poorly defined timing. ERDW asks whether that burden is present and whether bound communication can reduce it without harm. Fear of cancer recurrence describes what is feared. Intolerance of uncertainty explains why an unresolved threat can be difficult to tolerate. Scanxiety describes distress around scanning (Wroot et al., 2020; Russell et al., 2024; Tutelman and Heathcote, 2020; Tutelman et al., 2022; Cunningham et al., 2021; Pizzo et al., 2024; Fardell et al., 2016; Carleton, 2016; Peikert et al., 2020; Peikert et al., 2022; Bui et al., 2021; Heathcote et al., 2022). These literature justify the adjacent questions; they do not by themselves establish ERDW. ERDW asks whether the way a result pathway orders time, route, and explanation is associated with increased in burden during a defined event period compared with the inter-event baseline.

This is the proposed contribution to psycho-oncology. Fear of recurrence, scanxiety, and intolerance of uncertainty remain essential because they identify threat, event-related distress, and vulnerability. However, they do not by themselves make the temporal arrangement of care visible as a possible source of burden. ERDW makes that arrangement open to inquiry: the waiting window, the promised or unpromised reply, the portal release, the missed call, and the unanswered question of what delay means. Some survivors or parents may have high fear of recurrence between appointments and need sustained psychosocial support, even when no event-linked peak occurs. Others may have low background distress but still be disrupted by a brief, poor bounded result interval. ERDW should therefore remain a candidate-pathway filter rather than a new latent psychological entity.

Table 1 makes this conceptual positioning explicit. It does not suggest that ERDW replaces existing constructs. The columns distinguish the lens used, the question that lens primarily addresses, its explanatory value, and the operational contribution ERDW adds. Fear of cancer recurrence specifies the threat; scanxiety anchors distress in surveillance imaging; intolerance of uncertainty explains vulnerability to unresolved threat; and the work on waiting and temporal disruption explains how time can become burdensome. ERDW draws on these perspectives but adds a narrower service-design question: whether the timing and route of result communication produce a measurable event-linked deviation from baseline burden.

**Table 1:** Conceptual distinction and operational contribution of ERDW.

Lens	Core question	Primary explanatory strength	Specific ERDW contribution
Fear of cancer recurrence	What threat is feared?	Recurrence or progression threat, somatic worry, and child–parent measures.	Distinguishes sustained baseline burden from event-linked deviation.
Scanxiety	What happens around scans?	Imaging-related distress and repeated assessment around surveillance scans.	Uses imaging as the empirical anchor, without extending the claim to all result pathways.
Intolerance of uncertainty	Who is vulnerable to unresolved threat?	Trait/process vulnerability to uncertainty, checking, and worry.	Tests whether uncertainty is partly service-produced, not only person-held.
Waiting and temporal disruption	How does time become burdensome?	Waiting, suspended normality, and disruption of illness timelines.	Turns waiting from a narrative burden into a time-stamped service signal.

## Eligibility criteria for candidate pathways

The four eligibility criteria are not a theory. They should not be read as necessary or sufficient conditions for distress. They are a first act of ordering: a way to decide which pathways deserve study before every instance of waiting becomes a research object. The filter rests on four premises. Uncertainty requires an outcome or timing that is unresolved. Waiting becomes harder when perceived consequences matter. Checking becomes possible when a contact route or portal exists. Service evaluation is justified only when the pathway is time-stamped and modifiable.

A pathway is suitable for first testing when four conditions are met. It is scheduled or time-stamped; it includes uncertainty about outcome or timing; it carries perceived consequences for recurrence, late effects, identity, or continuity of care; and it has a plausible route for checking or unscheduled contact. These criteria should be revised after co-design and early empirical work. Their value is practical. They identify service processes that can be measured and changed, rather than assuming that an ERDW already exists. They also limit how evidence should be used: studies of adjacent phenomena should be treated as conceptual or methodological support unless they directly address the candidate pathway being tested.

The evidence hierarchy should remain restrictive. Existing references support adjacent constructs and methods rather than ERDW itself. The closest empirical anchor is a smartphone ecological momentary assessment (EMA) feasibility study of scanxiety in 30 adolescent and young adult survivors. The study found high daily fear of recurrence and negative affect before surveillance scans than after them (Heathcote et al., 2022). This finding supports the time-resolved research question around imaging. It does not establish ERDW across pediatric results pathways or justify generalization to all follow-up events. Blood-test review is the nearest pragmatic analog, but only where timing, latency, and disclosure routes are consistent enough to map a credible waiting window. Portal release, letters, transition points, and anniversaries should remain exploratory boundary cases until their timing, latency, consequence, and disclosure pathways can be specified.

## Bounded communication as a message condition

Bounded result communication should be treated as a testable message condition, not as a ready intervention. The mechanism is intentionally small. Unclear timing or disclosure routes create temporal uncertainty. If that uncertainty is left unaddressed, worst-case inference, check, and vigilance may follow. These proximal processes may increase the burden of waiting. A bound message tests whether clear information about timing, route, portal visibility, delay, and contact changes those proximal outcomes.

The message condition can be described without turning it into clinical advice. Families would be told the expected result window, who will communicate results, which route will be used, whether results might appear in a portal before clinician interpretation, and what to do if the window is missed. The purpose is orientation by form, not reassurance by tone alone. These elements are author-derived and pre-co-designed. They should not be used as clinical material, recruitment wording, or an implementation template until survivors, parents, adolescents, multilingual families, and teams have tested their wording and burden.

First outcomes should remain close to this mechanism: waiting-related uncertainty, checking, sleep disruption, unscheduled contact, preparedness, and possible harm to salience or equity. Global quality of life may matter later. For a first signal question, however, it is too distant from the interval being tested.

## From perspective to evaluation

After defining ERDW and narrowing eligible pathways, the Perspective turns to evaluation. ERDW sharpens a service question; it is not a protocol in disguise. Before any pilot, services should map the result pathways, usual care, portal timing, delay practices, and communication problems defined by families themselves. This pre-feasibility step is especially important for non-imaging pathways. In those pathways, the waiting window may be less visible, more variable, and more dependent on local habit than on formal design.

The first empirical question should remain narrow. Do imaging result pathways and only carefully selected blood-test pathways with consistent latency and disclosure routes, show a time-linked signal relative to inter-event burden? A short event window can be used as a feasibility parameter, with timing adapted to imaging and blood-test pathways. The analysis plan should be specified with biostatistical input. It should address autocorrelation, missingness, prompt reactivity, and separation of event-window deviation from sustained baseline burden (Shiffman et al., 2008; Hamaker and Wichers, 2017).

Design details should remain illustrative at this stage. Cluster-period allocation may reduce contamination because communication practices operate at the team level. However, the number of clusters and periods, retention assumptions, and uncertainty around intra-cluster correlation belong in a protocol rather than in this Perspective (Whitehead et al., 2016; Cocks and Torgerson, 2013; Eldridge et al., 2016).

Table 2 is therefore a decision roadmap, not a study protocol. Each row marks a point at which the ERDW frame must be specified, modified, or abandoned. The sequence moves from candidate pathway selection to co-design, message specification, measurement, and interpretation. The final column is deliberately included to prevent implementation drift: if the waiting window cannot be mapped, if families or teams reject the assumptions, if the message increases burden, or if measurement produces the signal it seeks to observe, the ERDW frame should be narrowed or abandoned.

**Table 2:** Roadmap from ERDW perspective to first evaluation.

Step	Decision focus	Minimum specification	Narrow or abandon if
1. Candidate pathway	Which service process is worth testing?	Start with imaging; consider a blood test review only when timing, latency, and disclosure route are consistent.	No credible waiting window can be mapped.
2. Co-design	Would families accept the language and method?	Co-design terminology, message condition, EMA wording, adolescent safeguards, and non-portal routes.	Families, adolescents, multilingual groups, or teams reject these assumptions.
3. Message condition	What temporal ambiguity is being reduced?	Specify result window, communicator, route, portal expectation, delay plan, and contact route as the test condition.	The message increases salience, checking, burden, or exclusion.
4. Measurement	Can the signal be measured without producing it?	Use brief event-window assessment of uncertainty, checks, sleep, contact, preparedness, and harms.	Assessment is not tolerated, missingness is patterned, or prompts appear reactive.
5. Interpretation	What would count as useful?	Look for temporal concentration, mechanism-consistent change, and no added harm.	Deviation is absent, inseparable from the baseline burden, or inconsistent with event timing.

## Harms, equity, and children as actors

Harms should be measured as seriously as benefits. The risks are concrete. A message meant to reduce uncertainty may make the result more salient. A prompt intended to measure distress may provoke checking. A portal route meant to improve access may exclude families who cannot use it safely or confidently. Other risks include labeling harm, work-shift harm, EMA reactivity, dyadic spillover, and digital or language exclusion. Dyadic spillover includes the synchronized escalation of parent and survivor distress. It also includes family communication patterns in which one person’s checking, reassurance seeking, or avoidance amplifies another person’s burden.

Treating waiting time as a modifiable part of care therefore requires a clear ethical boundary. ERDW does not propose that services should manage a family’s subjective time, emotional pace, or domestic rhythm. Furthermore, it does not aim to completely control waiting time or eliminate all uncertainty. Its target is narrower: service-produced temporal ambiguity, such as uncertainty about when results can be expected, who will interpret them, which route will be used, or what a delay means. In this sense, time is addressed only insofar as it is structured by care; the purpose is to reduce avoidable ambiguity without colonizing family life or making surveillance more salient. Feasibility work should therefore assess temporal harm, including clock-watching, checking, anticipatory vigilance, disruption of family routines, prompt reactivity, or shifting responsibility for time management onto families.

Bounded communication must not default to portal-first communication. A pathway that reduces uncertainty for digitally confident families may increase it for families who cannot access, interpret, or emotionally tolerate portal-based results (Bhalla et al., 2024; Schultz et al., 2021; Sisk et al., 2023; Patel et al., 2025; Griffin et al., 2024; Ouellet et al., 2025). Message conditions should therefore be multilingual, literacy-sensitive, and available through non-portal routes. These routes may include interpreter-supported calls or translated written information.

Population differences should therefore be treated as design variables rather than background variation. Age and developmental stage may shape what information is understood, sought, avoided, or shared. Prior illness experiences, family communication patterns, language, health literacy, digital access, and local service context may also change how a waiting interval is experienced (Wroot et al., 2020; Russell et al., 2024; Tutelman and Heathcote, 2020; Tutelman et al., 2022; Cunningham et al., 2021; Pizzo et al., 2024; Fardell et al., 2016; Carleton, 2016; Peikert et al., 2020; Peikert et al., 2022; Bhalla et al., 2024; Schultz et al., 2021; Sisk et al., 2023; Patel et al., 2025; Griffin et al., 2024; Ouellet et al., 2025). Adults who survived childhood cancer, parents, adolescents, younger children, multilingual families, and families with limited digital access may therefore face different risks and forms of support. ERDW should not be read as a universal experience across groups. It is a candidate service-level frame that must be adapted and tested within the population and communication pathway in which it is used.

Older children and adolescents require particular attention because they are not passive recipients in result communication. They may receive, avoid, reinterpret, or share information differently from their parents. Young children may be affected more indirectly through parental interpretation, family routines, and parental distress. Proxy portal access, consent, data minimization, confidentiality, and local data protection laws, including GDPR where applicable, should therefore be treated as design variables. They should not be treated as background details.

## Patient and public involvement and co-design

There was no formal patient and public involvement for this Perspective. This limits the confidence in the acceptability of terminology, EMA domains, message elements, and assumptions regarding harm. Therefore, ERDW should be regarded as a pre-co-design.

To ensure that ERDW remains implementable, co-design should distinguish core components from adaptable delivery features. The core is not defined by fixed wording or a single communication channel. Instead, it is the presence of a time-stamped event, an unresolved result or timing question, a perceived consequence, and a plausible route for communication, checking, or unscheduled contact. For bounded communication, the corresponding core elements are the expected result window, the responsible communicator, the communication route, portal expectations, the delay plan, and the contact route.

The adaptable perimeter includes language, translation, reading level, age-appropriate wording, preferred routes, burden thresholds, and safeguards. These features should be documented as design variables rather than as uncontrolled variation. In this sense, co-design preserves comparability by testing the same minimal mechanism in locally acceptable forms.

Co-design is part of validity, not a dissemination step after the fact. Before conducting feasibility testing, survivors, parents, adolescents, multilingual families, and clinical teams should confirm whether the proposed core is acceptable and should shape the adaptive perimeter: the language of waiting, the message condition, delivery routes, EMA wording, burden thresholds, stop-or-revise criteria, and population-specific safeguards. The process should actively look for divergent needs across age, developmental stage, family role, prior illness experience, language, health literacy, digital access, and local care context. Transparent methods such as GRIPP2-informed reporting should be used (Staniszewska et al., 2017). Without this groundwork, the message condition will remain merely illustrative and not suitable for implementation.

## Discussion

Current survivorship and psycho-oncology literatures document late-effect surveillance needs, mental health surveillance needs, fear of recurrence, scanxiety, intolerance of uncertainty, medical waiting, EMA methods, feasibility standards, co-design reporting, and inequities in portal-based communication (Bhakta et al., 2017; Landier et al., 2018; Marchak et al., 2022; Mishel, 1988; Charmaz, 1983; Timmermans and Buchbinder, 2010; Wongsomboon and Shepperd, 2022; Wroot et al., 2020; Russell et al., 2024; Tutelman and Heathcote, 2020; Tutelman et al., 2022; Cunningham et al., 2021; Pizzo et al., 2024; Fardell et al., 2016; Carleton, 2016; Peikert et al., 2020; Peikert et al., 2022; Bui et al., 2021; Heathcote et al., 2022; Shiffman et al., 2008; Hamaker and Wichers, 2017; Whitehead et al., 2016; Cocks and Torgerson, 2013; Eldridge et al., 2016; Bhalla et al., 2024; Schultz et al., 2021; Sisk et al., 2023; Patel et al., 2025; Griffin et al., 2024; Ouellet et al., 2025; Staniszewska et al., 2017). These advances identify important burdens, concepts, methods, and safeguards. They do not yet specify whether the temporal structure of the result communication itself can be observed as a modifiable service signal. The clinical problem is therefore real, but the literature currently supports ERDW as a question to be tested rather than as an established empirical construct.

The contribution of this Perspective is threefold. First, it defines ERDW as a narrow service-design heuristic rather than a new distress label. Second, it specifies a minimal uncertainty mechanism that links unclear timing and route to worst-case inference, checking, vigilance, and waiting-related burden. Third, it turns that mechanism into a falsifiable service-level question: does a candidate result pathway produce a time-linked deviation from inter-event burden, and can bounded communication change proximal outcomes without harm?

This contribution should remain intentionally small. The strength of ERDW is that it moves a familiar psycho-oncology problem from a diffuse account of distress to a service-level question that can be specified, co-designed, and rejected if unsupported. Its limits are equally important. ERDW does not propose another distress label, claim that waiting can be fully controlled, or seek to manage families’ subjective time, emotional pace, or domestic rhythm. Its ethical object is service-produced temporal ambiguity, and not family life itself. ERDW should also not be universalized across survivors, parents, young children, adolescents, adults, or multilingual families. Each use requires population-specific co-design and feasibility testing. The conclusion is conditional: ERDW is worth pursuing only if burden concentrates in time beyond the inter-event baseline, fits a plausible uncertainty mechanism, and can be studied or addressed without adding salience, reactivity, inequity, temporal harm, or dyadic harm. If those conditions are not met, the frame should be narrowed, redirected to a more suitable pathway, or abandoned.

This is why the Perspective remains directed to a psycho-oncology readership. It asks not only which families are distressed while waiting but also which result-communication pathways leave uncertainty unnecessarily unbounded. This service-level question connects psychological burden, survivorship communication, and ethical service design, highlighting how the article fits within the psycho-oncology section.

## Data Availability

The original contributions presented in the study are included in the article/supplementary material, further inquiries can be directed to the corresponding author.
